# Supplementary material for: The Effect of Long Chain n-3 Fatty Acid Supplementation on Muscle Strength in Older Adults: A Systematic Review and Meta-Analysis
Source: Nutrients. 2023 Aug 14;15(16):3579. doi: 10.3390/nu15163579 (PMC10458650; doi:10.3390/nu15163579)
Supplement: Supplementary file 1 [file nutrients-15-03579-s001.zip › nutrients-2491497-supplementary.pdf]

Supplementary Table S1. Keyword and search terms

Search Number	Search Term
1	"Older adult"[Mesh] OR elderly OR older OR older adults OR elderly OR senior OR senior citizens OR aging population OR elder OR aged
2	"Fish oil"[Mesh] OR “Krill oil” OR “Omega-3 fatty acids” OR “n3 PUFA” OR “DHA” OR “EPA”
3	"Muscle AND mass” OR “lean"[Mesh] OR volume” OR “grip*” OR “strength” OR “fat free*” OR “DXA” OR “DEXA*”
4	1 AND 2 AND 3

Supplementary Table S2. Participant, intervention, comparison, outcome, and study design (PICOS) with inclusion and exclusion criteria to determine study eligibility.

Review Questions	Inclusion criteria	Exclusion criteria
Population (P)	Healthy adults with mean sample age 65 years of age or over.	Participants who had major chronic disease (eg cancer, kidney disease, liver disease, diabetes mellitus and cardiovascular disease, uncontrolled hypertension).
Intervention (I)	LCn-3 PUFA (EPA/DHA) supplementation, such as fish oil or krill.	Other interventions, such as exercise or other supplements, included.
Comparator (C)	A control oil, such as vegetable oil or olive oil.	No control oil
Outcomes (O)	Measurement of muscle mass and muscle strength, with data on and muscle function also extracted.	
Study design	Randomised control trials (RCTs).	Any other study design
Other	Published peer-reviewed articles in scientific journals, in an English language.	

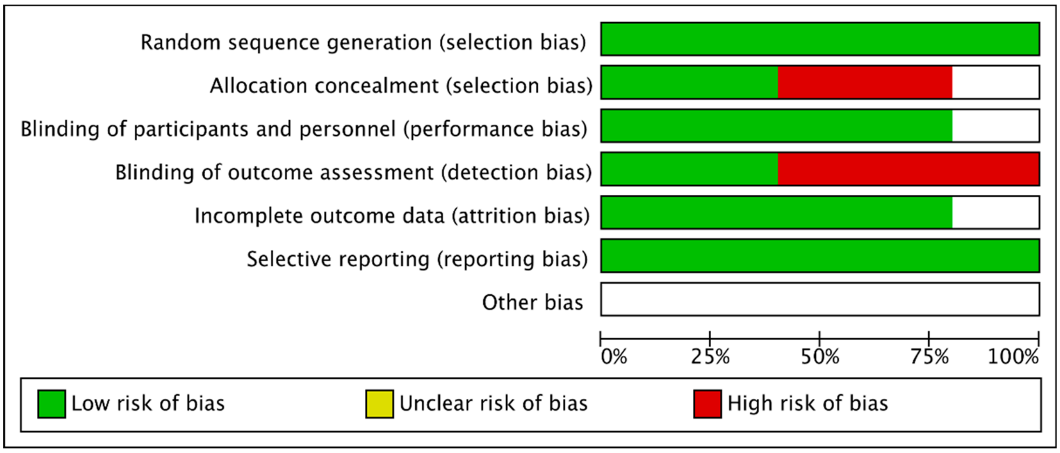

Supplementary Figure S1. A Summary of the risk-of-bias for all the included studies.

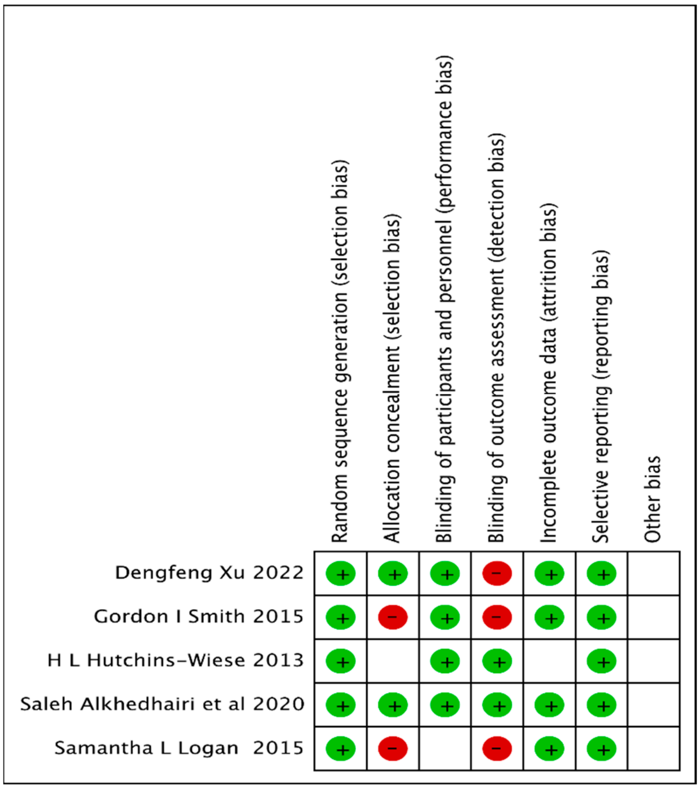

Supplementary Figure S2. Risk of bias assessment for the studies included [24-28].

Certainty assessment							№ of patients		Effect	Certainty
№ of studies	Study design	Risk of bias	Inconsistency	Indirectness	Imprecision	Other considerations	krill oil	placebo	Absolute-Relative (95% CI)	
**Hand Grip Strength (follow-up: range 12 weeks to 24 weeks)**										
5	randomised trials	not serious	serious	not serious	serious	publication bias strongly suspected	275	213	SMD 0.61 SD higher (-0.05 lower to 1.27 higher)	⨁◯◯◯ Very low

CI: confidence interval; SMD: standardised mean difference.

Supplementary Figure S3. The evidence certainty for the effects of LCn-3 PUFA supplements on hand grip strength was rated very low according to GRADE
